# Editorial: The force-velocity relationship: Assessment and adaptations provoked by exercise, disuse and disease

**DOI:** 10.3389/fphys.2022.1062041

**Published:** 2022-10-21

**Authors:** Julian Alcazar, Robert Csapo, Luis M. Alegre

**Affiliations:** ^1^ GENUD Toledo Research Group, Universidad de Castilla-La Mancha, Toledo, Spain; ^2^ CIBER on Frailty and Healthy Aging, Instituto de Salud Carlos III, Madrid, Spain; ^3^ Centre for Sport Science and University Sports, University of Vienna, Vienna, Austria

**Keywords:** force-velocity, load-velocity, torque-velocity, muscle, physiology

## Introduction

The slower a skeletal muscle shortens, the greater the force it can generate during contraction and *vice versa*. The literature shows that the force-velocity (F-V) relationship has important implications for different aspects of muscle and exercise physiology, such as muscle efficiency and fatigability, the assessment of adaptations provoked by exercise training, as well as the understanding of the pathophysiology of several myopathies or the mechanisms of muscle contraction *per se* (Alcazar et al., 2019). Also, it may be of relevance for other fields of research, such as robotics or the development of protheses featuring natural muscle-like properties (Schmitt et al., 2012). In addition, the F-V relationship is frequently measured as indicator of physical performance in sports and functional health in the general population. The main goal of this Research Topic was to encourage and collect studies addressing: the optimization of the assessment and analysis of the F-V relationship; the effects of exercise, disuse and disease on the F-V relationship; and the significance of the F-V relationship for physical performance in sports, activities of daily living and health.

## Molecular insights on the F-V relationship

The shape of the force-velocity relationship in skeletal muscles is determined by the molecular mechanisms that yield muscle contractions, making Huxley’s theory of muscle contraction (Huxley, 1957) and Hill’s equation for modeling the F-V relationship (Hill, 1938) not only compatible but inherently linked. The work by Seow and Seow summarizes the molecular events and changes that shape the F-V relationship of activated muscle. Thus, for example, the authors expose the influence that changes in muscle activation or muscle hypertrophy, temperature, or different muscle characteristics have on the shape of the F-V relationship. It is also of interest to realize how the normalized F-V relationship of skeletal muscle can be used to ascertain the molecular events leading to the crossbridge cycle Seow and Seow.

## The assessment of the F-V relationship

When deciding how to assess the F-V relationship in exercising humans there are many variables that can be chosen, among others, the type of exercise, the number of trials or the mathematical model.

Smajla et al. compared the F-V profiles obtained during flywheel squats vs. countermovement jumps. The evidence, in conjunction with the rest of the literature, points out that the main outcomes provided by the F-V relationship are not interchangeable when the differences between the selected exercises are remarkable (e.g., resistance mode). In this case, the decision should be made based on the main goal of the assessment, or on the relationship that it has with other outcomes (e.g., sport performance). On the other hand, the F-V relationship in other exercises that may provide distinct information, as those targeting the core muscles, has been poorly investigated, as noted in the scoping review conducted by Zemková. Of note, these muscles are considered relevant for stabilization of the spine and production of high force in different everyday- and sport-related physical activities Zemková. Regarding the equation chosen to model the F-V relationship, curvilinear models seem to fit registered F-V data better than linear models. However, it has been showed that F-V data deviate from the traditional rectangular hyperbola at both ends of the relationship (Alcazar et al., 2019), and so other models have been recently proposed in the literature (Alcazar et al., 2022). Even so linear models may still be valid and helpful in some cases. Sašek et al. found that the use of the linear 2-point method is also valid for testing isokinetic knee extension and flexion function, especially when using two distant F-V points and excluding very light intensities, since the latter deviate from linearity.

## Association between the F-V relationship and physical performance

The association between the outcomes derived from the F-V relationship and physical performance is clear (Baena-Raya et al., 2022). In that sense, Smajla et al. compared the advantage of assessing the F-V relationship during countermovement jumps vs. flywheel squats in elite karatekas. Specifically, high force and power were positively correlated to physical performance; however, while the F-V relationship obtained during countermovement jumps was better correlated with the karateka’s agility performance than the one obtained during flywheel squats, while the opposite occurred for the correlation to a short karate specific test Smajla et al.

## Limitations and perspectives

Despite the widespread interest in the F-V relationship (or its proxies, such as the load-velocity and torque-velocity relationships), there are still some unresolved questions regarding its assessment and interpretation. For example, there are no specific guidelines on how the F-V relationship should be objectively measured. Also, it is poorly understood how the F-V relationship and its normalized curve can be specifically modulated by exercise training in different populations, and changes occurring during growth, aging and disease have not been investigated yet. A special mention should also be made to the lack of studies on the F-V relationship conducted in female participants. Therefore, it is also our goal to encourage research addressing these gaps in the literature, even after this Research Topic has been closed.
